# Aberrant microbiomes are associated with increased antibiotic resistance gene load in hybrid mice

**DOI:** 10.1093/ismeco/ycae053

**Published:** 2024-04-15

**Authors:** Víctor Hugo Jarquín-Díaz, Susana Carolina Martins Ferreira, Alice Balard, Ľudovít Ďureje, Milos Macholán, Jaroslav Piálek, Johan Bengtsson-Palme, Stephanie Kramer-Schadt, Sofia Kirke Forslund-Startceva, Emanuel Heitlinger

**Affiliations:** Max-Delbrück-Center for Molecular Medicine in the Helmholtz Association (MDC). Robert-Rössle-Str. 10, 13125 Berlin, Germany; Leibniz Institute for Zoo and Wildlife Research (IZW), Alfred-Kowalke-Straße 17, 10315, Berlin, Germany; Experimental and Clinical Research Center, a cooperation between the Max-Delbrück-Center for Molecular Medicine in the Helmholtz Association and the Charité–Universitätsmedizin, Berlin, Germany; Charité – Universitätsmedizin Berlin, corporate member of Freie Universität Berlin and Humboldt-Universität zu Berlin, Experimental and Clinical Research Center, Lindenberger Weg 80, 13125, Berlin, Germany; Department of Molecular Parasitology, Institute for Biology, Humboldt University Berlin (HU). Philippstr. 13, Haus 14, 10115, Berlin, Germany; Department of Molecular Parasitology, Institute for Biology, Humboldt University Berlin (HU). Philippstr. 13, Haus 14, 10115, Berlin, Germany; Division of Computational Systems Biology, Center for Microbiology and Ecological System Science, University of Vienna, Djerassipl. 1, 1030, Vienna, Austria; Leibniz Institute for Zoo and Wildlife Research (IZW), Alfred-Kowalke-Straße 17, 10315, Berlin, Germany; Department of Molecular Parasitology, Institute for Biology, Humboldt University Berlin (HU). Philippstr. 13, Haus 14, 10115, Berlin, Germany; Research Facility Studenec, Institute of Vertebrate Biology, Czech Academy of Sciences, Květná 8, 60365, Brno, Czech Republic; Laboratory of Mammalian Evolutionary Genetics, Institute of Animal Physiology and Genetics, Czech Academy of Sciences, Veveri 97, 60200, Brno, Czech Republic; Research Facility Studenec, Institute of Vertebrate Biology, Czech Academy of Sciences, Květná 8, 60365, Brno, Czech Republic; Division of Systems and Synthetic Biology, Department of Life Sciences, SciLifeLab, Chalmers University of Technology, Kemivägen 10, SE-412 96, Gothenburg, Sweden; Department of Infectious Diseases, Institute of Biomedicine, The Sahlgrenska Academy, University of Gothenburg, SE-413 46, Gothenburg, Sweden; Centre for Antibiotic Resistance Research (CARe) in Gothenburg, Sweden; Leibniz Institute for Zoo and Wildlife Research (IZW), Alfred-Kowalke-Straße 17, 10315, Berlin, Germany; Institute of Ecology, Technische Universität Berlin, Rothenburgstr. 12, 12165, Berlin, Germany; Max-Delbrück-Center for Molecular Medicine in the Helmholtz Association (MDC). Robert-Rössle-Str. 10, 13125 Berlin, Germany; Experimental and Clinical Research Center, a cooperation between the Max-Delbrück-Center for Molecular Medicine in the Helmholtz Association and the Charité–Universitätsmedizin, Berlin, Germany; Charité – Universitätsmedizin Berlin, corporate member of Freie Universität Berlin and Humboldt-Universität zu Berlin, Experimental and Clinical Research Center, Lindenberger Weg 80, 13125, Berlin, Germany; German Centre for Cardiovascular Research (DZHK), Partner Site Berlin, Berlin, Germany; Leibniz Institute for Zoo and Wildlife Research (IZW), Alfred-Kowalke-Straße 17, 10315, Berlin, Germany; Department of Molecular Parasitology, Institute for Biology, Humboldt University Berlin (HU). Philippstr. 13, Haus 14, 10115, Berlin, Germany

**Keywords:** antimicrobial resistance gene, microbiome, hybridization, mice

## Abstract

Antibiotic resistance is a priority public health problem resulting from eco-evolutionary dynamics within microbial communities and their interaction at a mammalian host interface or geographical scale. The links between mammalian host genetics, bacterial gut community, and antimicrobial resistance gene (ARG) content must be better understood in natural populations inhabiting heterogeneous environments. Hybridization, the interbreeding of genetically divergent populations, influences different components of the gut microbial communities. However, its impact on bacterial traits such as antibiotic resistance is unknown. Here, we present that hybridization might shape bacterial communities and ARG occurrence. We used amplicon sequencing to study the gut microbiome and to predict ARG composition in natural populations of house mice (*Mus musculus*). We compared gastrointestinal bacterial and ARG diversity, composition, and abundance across a gradient of pure and hybrid genotypes in the European House Mouse Hybrid Zone. We observed an increased overall predicted richness of ARG in hybrid mice. We found bacteria–ARG interactions by their co-abundance and detected phenotypes of extreme abundances in hybrid mice at the level of specific bacterial taxa and ARGs, mainly multidrug resistance genes. Our work suggests that mammalian host genetic variation impacts the gut microbiome and chromosomal ARGs. However, it raises further questions on how the mammalian host genetics impact ARGs via microbiome dynamics or environmental covariates.

The rise of antibiotic-resistant bacteria and the evolution and spread of antimicrobial resistance genes (ARGs) are major global health concerns [1]. Indiscriminate antimicrobial use in clinical and veterinary settings exacerbates the problem. Although wildlife’s role as a reservoir for zoonotic pathogens is established, their contribution to antimicrobial resistance spread is understudied [2]. The external environment, diet, and mammalian-host phylogeny shape the microbiomes of wildlife [3]. ARGs spread and evolve in nested environments within animal and bacterial host communities. However, whether these factors, especially host genetics, directly or indirectly impact ARGs in wildlife populations remains unclear.

Rodents, particularly synanthropic house mice, carry and potentially disseminate ARGs [4]. House mice, with genetically diverse subspecies and microbiome variations [5, 6], are a suitable model to investigate the effects of host genetics and bacterial community composition on ARGs. Hybrids between these subspecies show much more extreme high or low infection loads of eukaryotic parasites [7, 8] and viruses [9] as well as the composition of the microbiome [10] and fungi [11] than pure subspecies, and are referred to as “transgressive phenotypes.” We studied the European House Mouse Hybrid zone (HMHZ), a semipermeable barrier between *Mus musculus musculus* and *Mus musculus domesticus*, to assess the impact of this barrier and hybridization on ARGs through microbiome selection.

We analysed the colon content microbiome of 493 wild mice from 160 trapping localities in two different geographical transects across the HMHZ, one in northeastern Germany close to Berlin (Brandenburg, *N =* 441) and a second one in southeastern Germany close to the Czech border (Bavaria, *N* = 52) (Fig. 1A, Supplement 1). We found that neither locality nor year of collection were significant predictors of the microbial composition (Beta diversity), while hybridicity explained a low but significant proportion of the overall microbial compositional variance while adjusting for the spatial effects of locality (Permutational multivariate analysis of variance (PERMANOVA), *F* = 1.38, df = 1, R^2^ = 0.003, *P* = .047) and geographic distance (Supplement 2). Differences in the microbiome of the subspecies and microbiome disruption in hybrids have been observed previously [10, 11]. This finding might reflect the association between bacteria and host genetics [12]. It also raises the question of whether the genetic differentiation of hosts and hybridization influences antibiotic resistance toward a human-relevant microbiome phenotype.

**Figure 1:**
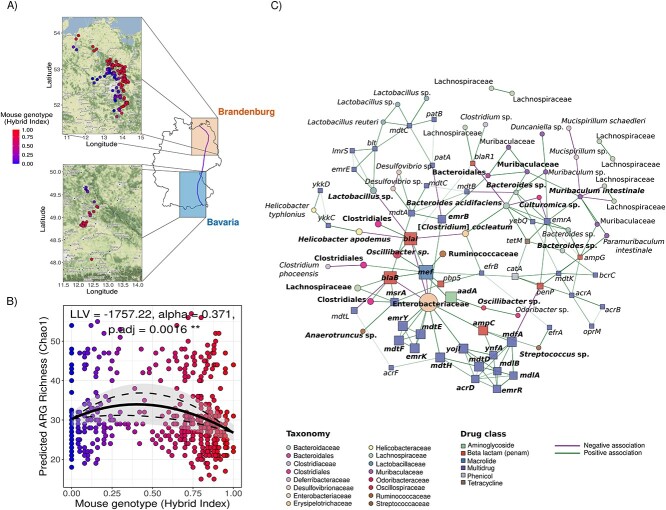
Antibiotic resistance and regulatory genes associated with ARGs in house mouse microbiome; (A) geographical distribution of collected house mice included in this study; the colon content microbiome was assessed for 493 house mice collected from two different transects, in northeastern Germany close to Berlin (Brandenburg, *N =* 441) and southeastern Germany close to the Czech border (Bavaria, *N* = 52), both along the HMHZ (schematized by the purple line); the map on the right-side of the panel indicates the approximate location of both transects along the hybrid zone (purple); each point represents a mouse; the scale in the x-axis indicates the genotype of the mouse (hybrid index: HI), ranging from pure *M. m. domesticus* (HI = 0.0, *N* = 79) to pure *M. m. musculus* (HI = 1.0, *N* = 23); (B) hybrid effect on ARG richness is independent of the transect; ARG richness predictions were compared across a gradient of *Mus musculus* genotypes (HI), ranging from 0 (pure *M. m. domesticus*, in blue) to 1 (pure *M. m. musculus*, in red), to (i) test hybrid effect on alpha diversity and (ii) detect differences on alpha diversity between parental subspecies, transects or both; the richness of predicted ARGs increased towards the centre of the hybrid zone, supporting a hybrid (transgressive) effect on the richness of ARGs (Chao1 index, LL = −1757.22, α= 0.371, *padj =* .0015); ARG content in the parental subspecies *M. m. domesticus* was richer than *M. m. musculus* parental subspecies (*G* test: χ^2^(2, 493) = 4.74, *padj* = .007); (C) house mouse network analysis on the co-abundance of ARG and bacterial ASV abundance; nodes correspond to ASVs or ARGs, and colours correspond to the annotated family or target drug class category; node size was scaled based on Kleinberg’s hub scores (hub centrality scores); the metric increases with the number of links a node has to other nodes and with the relevance in the structure of the network (higher if a node is connecting different clusters of the network); high hub centrality scores reflect higher influence of a node on other taxa and ARGs; edges are predicted interactions, green edges are positive and purple are negative, and edge thickness reflects association strength; bacterial nodes are circles, and ARGs are squared; in bold, ASVs and ARGs with the highest hub scores.

To assess whether hybridization also affects the occurrence and abundance of ARGs, we predicted the ARGs content from the bacterial chromosomal genome but not mobile genetic elements (e.g. plasmids) based on 16S rRNA gene amplicon data (Supplement 3) [13]. The ARG content included genes involved in antibiotic resistance phenotype and regulatory genes associated with ARGs. We found multidrug resistance proteins and multidrug efflux pumps to be the most prevalent ARG class, accounting for 54.3% of the overall ARG content, followed by genes involved in beta-lactam alteration and inactivation (32.4%), resistance genes for phenicol (6.54%), macrolides (3.85%), and tetracycline (2.83%) antibiotics. The most abundant gene was *acrA* (16.02%), encoding a subunit of the multidrug efflux complex AcrA-AcrB-TolC. Other ARGs relevant to environmental surveillance [14], including *blaR1*, *mdtK*, *catA*, *penP*, and *tetM*, had relative abundances of ~2.5% each in our mouse host microbiome.

Most strikingly, we observed a significant increase in the predicted ARG richness in hybrid mice (Log-Likelihood = −1757.22, transgressiveness [α] = 0.371, *padj =* .0016) (Fig. 1B) and interpreted this as a transgressive phenotype [6, 7]. The hybrid effect was robust when tested with additional alpha diversity indices (Supplement 4). In addition to hybrids, the parental subspecies *M. m. domesticus* also had a richer ARG repertoire than *M. m. musculus* (*G* test: χ^2^(2, 493) = 4.74, *padj* = .007). At the same time, the hybrid effect was only observed for bacteria richness in the Bavarian transect (Supplement 4). ARG richness was strongly shaped by localities and abundance of relevant ARG-associated phyla (e.g. *Proteobacteria*) (Analysis of Variance (ANOVA) alpha diversity) (Supplement 5). Hybridicity was also a significant predictor for ARG richness (ANOVA *F* = 7.89, df = 1, R^2^ = 3.41, *P* = .005; Supplement 5) and composition (PERMANOVA, *F* = 4.064, df = 1, R^2^ = 0.008, *P* = .012; Supplement 6), meaning that more and different ARGs were found in hybrid mice. An overall increased richness of ARGs in hybrids could be due to the disruption of the microbiome composition. The aberrant microbiomes, with transgressive microbial abundance, in hybrids represent less complex communities, potentially promoting selection for resistance. Complex microbial communities substantially decrease selection for resistance in semi-natural microbial communities exposed to antibiotics. Thus, interspecific competition within the microbiome could explain low resistance levels in naturally more complex bacterial communities [15].

We used a co-abundance network to explore the interaction among ARGs and bacterial taxa and investigated whether these hub taxa and ARGs showed transgressive phenotypes directly. We obtained a modular network and identified 91 bacterial hosts for 48 ARGs (Fig. 1C). We tested for hybrid effect (if the trait is transgressive) on the abundance of the 20 bacterial amplicon sequence variants (ASVs) and ARGs most relevant for community composition and with potential ecologically relevant associations (by Kleinberg’s hub centrality scores). Bacteria and ARG transgressiveness were not linked to their prevalence. While one ASV belonging to the family *Enterobacteriaceae* and one to the genus *Streptococcus* were transgressive with lower abundance in hybrids, *Muribaculum intestinale* had a higher abundance in hybrids (Fig. 2). At the ARG level, 15 multidrug efflux pumps with strong centrality were impacted by hybridization (Fig. 2).

**Figure 2:**
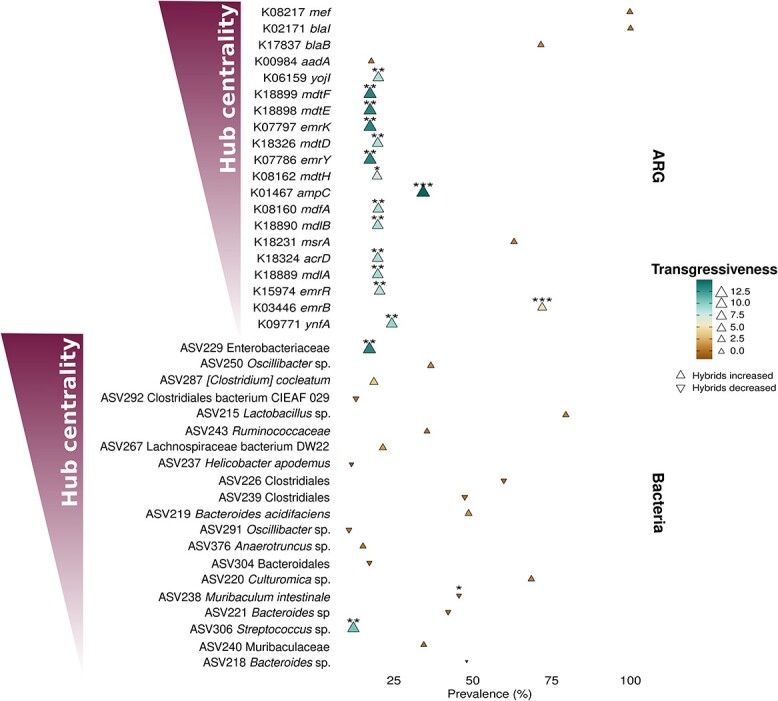
Transgressive abundance patterns in central antimicrobial resistance genes and bacteria for community composition; ARGs and bacterial ASVs are listed in decreasing order based on Kleinberg’s hub centrality scores (gradient of hub centrality, left); the prevalence for ASVs and ARGs is represented in the *x-*axis; the hybrid effect on the abundance of bacteria, or ARGs (transgressiveness), is encoded in the size and colour, its direction in shape; most important (central) ARGs are transgressive, showing an increased abundance in hybrids and are multidrug resistance genes; *P*-values were adjusted for false discovery rate (FDR) employing the Benjamini–Hochberg procedure; FDR-values <0.001 = ***, <0.01 = **, <0.05 = *.

Most ARGs with increased abundance in hybrids were multidrug resistance genes (Fig. 2), highly conserved among bacterial species [16]. Their relevance in clinically acquired resistance is usually low since they are chromosomally encoded and rarely transmitted among bacteria by horizontal gene transfer mechanisms (reviewed in Poole [17]). However, overexpression of multidrug efflux pumps has been linked to physiological advantages for resistant strains compared to sensitive ones [18]. Moreover, multidrug efflux determinants might be involved in self-protection systems against antibiotic-producing microbes (e.g. *Streptomyces* spp.) or other environmental stressors [19]. Thus, multidrug efflux determinants represent an initial intermediate resistance phenotype that may predict strains likely to evolve resistance, as shown in specific bacterial species [20, 21].

Our observations rely on predicted gene content, particularly chromosomal-encoded ARGs, which may have limitations [22]. However, our ARG predictions showed general congruence to ARG profiles at colon content based on metagenomic data (Supplement 7). Further studies and additional methods (qPCR or shotgun metagenomics) are needed to detect and confirm the resistome in natural house mouse populations and investigate the dynamics of ARG transmission within microbial communities. However, our findings emphasize the role of host genetic variation in shaping the gut microbiome and ARGs and provide the background to study the eco-evolutionary mechanisms of antimicrobial resistance emergence and transmission beyond the host genetic effect. House mice offer a suitable system for investigating bacteria–bacteria, host–gut microbiota, and host–landscape interactions, e.g., in a heterogeneous environment or different transects (Supplement S1.1) through extensive functional profiling and metagenomic analyses.

## Supplementary Material

Supplementary_material_Accepted_ycae053

## Data Availability

The scripts for the bioinformatic and statistical pipeline are available at https://git.bihealth.org/jarquivh/wildrodents_arg in a non-static version. Amplicon sequence data are deposited in the Short Read Archive (SRA) under project accession number PRJNA912123.
