# Supplementary material for: Aberrant microbiomes are associated with increased antibiotic resistance gene load in hybrid mice
Source: ISME Commun. 2024 Apr 15;4(1):ycae053. doi: 10.1093/ismeco/ycae053 (PMC11128261; doi:10.1093/ismeco/ycae053)
Supplement: Supplementary_material_Accepted_ycae053 [file supplementary_material_accepted_ycae053.docx]

############################**SUPPLEMENTS** ##############################

**Aberrant microbiomes are associated with increased antibiotic resistance gene load in hybrid mice**

Jarquín-Díaz, V. H., Ferreira, S. C. M., Balard, A., Ďureje, Ľ., Macholán, M.8, Piálek, J., Bengtsson-Palme J., Kramer-Schadt, S., Forslund-Startceva, S.K., Heitlinger, E.

**Supplement 1. Extended material and methods**

***S1.1 Collection of samples***

A total of 493 house mice were cross-sectionally sampled and used in the current study. Mice were trapped from farms and private facilities between 2015 and 2018 within the German federal states of Mecklenburg-Vorpommern, Bavaria, and Brandenburg (capture permit No. 2347/35/2014) from 160 trapping localities in two different geographical transects across the House Mice Hybrid Zone, one in northeastern Germany close to Berlin (Brandenburg, *N=* 441) and a second one in southeastern Germany close to the Czech border (Bavaria, *N*= 52) as described before (Jarquín-Díaz et al., 2019; Balard et al., 2020). While both trapping areas share similar climate conditions with continental weather, during the collection session in September, Brandenburg is slightly warmer and dryer (Temp: 15.1°C, 77% Rel. humidity, 1.46 mm/day rain) than Bavaria (Temp. 13.4°C, 79% Rel. humidity, 2.35 mm/day rain). Brandenburg’s landscape is highly fragmented and characterised by croplands for intensive agriculture and a minority of small scale farming. Bavaria’s landscape has almost half of the surface used for agriculture and one third for forestry. In brief, mice were trapped at the end of summer in September, ensuring the capture of a high number of individuals. Traps were set overnight per locality. Mice were housed individually in cages overnight and euthanized by cervical dislocation. All mice were dissected within 24 h after capture. Digestive tracts were dissected and colon content was snap-frozen in liquid nitrogen and then stored at −80° until further analysis.

***S1.2 Samples processing***

Colon content DNA was extracted using the NucleoSpinⓇ Soil kit (Macherey-Nagel GmbH & KG, Düren, Germany) following the instructions from the manufacturer. The quality and concentration of the extracts was determined by spectrophotometric measurements in a NanoDrop 2000c (Thermo Scientific, Walham, USA) and fluorometrically with Qubit dsDNA broad range assay kit (Thermo Scientific, USA), respectively. DNA extracts were normalised to a concentration of 30 ng/µL for further analysis.

Microfluidic PCR amplification of the V4 (~300 bp) hypervariable region of the 16S rRNA gene was done using the primers 515F-Y and 806R described by Parada et al., 2016 and Apprill et al., 2015, respectively. Universal adaptor sequences were added to the primer sequences for indexing.

Forward 515F-Y: [***ACACTGACGACATGGTTCTACA***]GTG**Y**CAGCMGCCGCGGTAA

Reverse 806R: [***TACGGTAGCAGAGACTTGGTCT***]GGACTAC**N**VGGGTWTCTAAT.

Microfluidic PCR amplification was performed with the FastStart™ High Fidelity PCR System, dNTPack (Sigma-Aldrich) in 5 μL final volume of reaction with primers at a final concentration of 250 nM and 10 ng/μL of extracted DNA under the following conditions: 50° for 2 min, 70° for 20 min, 95° for 10 min followed by 10 cycles of 95° for 15 s, 60° for 30 s, 72 °C for 1 min, two cycles 95° for 15 s, 80° for 30 s, 60° for 30 s, 72 °C for 1 min, eight cycles of 95° for 15 s, 60° for 30 s, 72 °C for 1 min, two cycles 95° for 15 s, 80° for 30 s, 60° for 30 s, eight cycles of 95° for 15 s, 60° for 30 s, 72 °C for 1 min and a finally five cycles 95° for 15 s, 80° for 30 s, 60° for 30 s in a FC1 Cycler (Fluidigm, USA). PCR amplicons were cleaned using the magnetic beads AMPure XP Reagent beads (Beckman Coulter Life Sciences, Krefeld, Germany), following the instructions of the manufacturer and eluted in 40 μL of elution buffer (10 mM Tris pH 8.5). Samples were randomised, including non-template controls (allowing detection of contaminant DNA introduced during library preparation and PCR amplification). Negative controls were processed and sequenced alongside the biological samples. Indexing was performed by a second PCR using 5 μL of the purified PCR products employing Access Array indexing primers (Fluidigm, USA). The second PCR was run at 95° for 3 min followed by 8 cycles of 95° for 30 s, 60° for 30 s, 72° for 30 s and a final extension at 72° for 10 min. Indexed amplicons were purified with magnetic beads and quantified using a Qubit 2.0 fluorometer with the dsDNA high sensitivity assay kit (Thermo Scientific, USA). Libraries were created by pooling each sample in equimolar concentrations. Quality and integrity of the final library was verified using the Agilent 2200 TapeStation with D1000 ScreenTapes (Agilent Technologies, USA). The pooled library was sequenced at the Berlin Centre for Genomics and Biodiversity Research (BeGenDiv) on the Illumina MiSeq platform using MiSeq v2 (500 cycles) reagent kit for 2 x 250 bp paired-end reads.

***S1.3 Sequence analysis***

Sequencing reads processing, from quality control to taxonomic assignment of amplicon sequence variants (ASVs) was performed using the DADA2 pipeline in R (v1.18) (Callahan et al 2016). In brief, both sequencing reads, forward and reverse, were trimmed to a length of 240 bp., allowing a maximum error of 2 nucleotides and zero ambiguous nucleotides (maxN=0, maxEE=2, truncQ=2). Taxonomy was assigned to the inferred ASVs using BLAST+ under an unbiased reference assignment to the non-redundant GenBank database. Assigned ASVs taxonomy, ASVs abundance and sample metadata were compiled into a single object using the package phyloseq v1.34.0 (McMurdie and Holmes, 2013). Before data analysis, an additional filter to eliminate 1) ASVs not assigned as Bacteria or "uncharacterized" ASVs at Phylum level and 2) low prevalent ASVs (less than ten samples, ~ 1.75%) was applied. To estimate alpha diversity metrics (observed richness, Chao1, Shannon, and Simpson indices) the dataset was rarefied to a threshold of 500 read depth based on rarefaction curves to ensure saturation of at least 70% of the samples. All further analyses employed the samples passing this pre-processing.

***S1.4 ARG prediction***

PICRUSt2 (Phylogenetic Investigation of Communities by Reconstruction of Unobserved States) pipeline v2.5.1 (Douglas et al., 2020) was used to infer the functional metabolic profiles of the bacterial communities of house mice microbiomes. This pipeline uses the 16S rRNA taxonomic profiles (ASVs) to predict functional gene families and abundance normalised by 16S copy numbers within a sample based on the Kyoto Encyclopaedia of Genes and Genomes (KEGG) abundances per sample. To determine the predicted “resistome”, consisting of ARGs involved in antibiotic resistance and regulatory genes associated with ARGs, hereafter referred only as ARGs, were subsetted from the predicted functional profile using an inclusion list with 103 KEGG orthologs (KO) numbers as described in the CARD database. ARGs or ARG cluster abundance is expressed as KO counts normalised by predicted 16S copy number abundances.

***S1.5 Diversity, composition and statistical analysis***

Host genotype was measured in two ways: hybrid index (HI) and hybridicity. HI represents the interspecific heterozygosity and is measured as the proportion of *Mus musculus musculus* alleles in a set of 14 diagnostic markers (Macholán et al., 2007; Baird et al., 2012) Based on their HI, we analysed pure *Mmd* (*N*= 79), pure *Mmm* (*N*= 23) and hybrid (*N*= 391)

mice. Hybridicity (absolute value of HI - 0.5) represents how close an individual is to a 50% contribution from both parental subspecies, ranging from 0 (pure hybrid genotype) to 0.5 (pure parental subspecies). This second measure allows analyses to be performed on a linear scale. To determine the relative influence of host and sampling derived factors on alpha diversity metrics, we used nested linear model comparison with richness (Chao1 index), diversity (Shannon index) and evenness (Simpson index) as response variables and hybridicity, year of sampling, and location of origin, bacterial richness, Firmicutes, Bacteroidetes and Proteobacteria abundance of as predictors. Models were compared by likelihood ratio test (LRT) to determine whether each parameter was significant. To determine the variation explained by each of the predictors in the linear models, we calculate the coefficient of determination (*R*^2^). All alpha diversity metrics were estimated based on the asymptotic rarefied ASVs and predicted ARGs. To test for the effects of host genetic (hybridicity) and temporal (year) and geographical (locality) variables on the bacterial and ARG community structure, we performed a permutational multivariate analysis of variance (PERMANOVA) on the bacterial and ARG beta-diversity. We used Bray-Curtis dissimilarity distances (999 permutations) implemented with the function adonis2 from the package vegan v2.5-7 (Oksanen et al. 2007) and we group for Locality within which to constrain permutations, while testing its relative impact on compositional changes.

***S1.6 Taxa-ARG co-abundance network analysis***

Co-abundance network analysis was used to identify: 1) potential ecologically relevant taxa-ARG associations and 2) individual taxa and ARG that significantly affect community structure. ASV and ARG abundance were filtered to 0.001% (at least 10 and 52 counts for ASV and ARG abundance, respectively) and to 10% prevalence (present in at least six samples) to reduce sparsity and ensure robustness. Co-abundance networks were created with the package SpiecEasi (Kurtz et al. 2015) using the “mb” neighbourhood selection method. We used the extended spiec.easi method for multiple microbial domains to construct the taxa-ARG network (Tipton et al. 2018). An optimal lambda coefficient was observed at 0.234 for the taxa-ARG network. Network and topological evaluations were done using *igraph* version 1.3.1 (Csardi and Nepusz, 2006). All analyses were done at ASV and ARG levels. We calculated modules based on the fast greedy modularity optimization algorithm (Clauset et al. 2004).

**S1.7 *Maximum likelihood analysis of non-linear effects of hybridisation***

We analysed hybridisation as a continuum using HI as a continuous predictor variable. We modelled the magnitude of the effect of hybridisation as a polynomial, estimating the degree to which new gene combinations are brought together (Baird et al., 2012; Balard et al., 2020). We maximise the likelihood of observed (richness or taxa intensity) data assuming either the same or different parameter values for pure parental subspecies (*Mmm* and *Mmd*) and either the presence or absence of this hybridisation effect (𝛼). As additional predictor variables we considered parental subspecies (*Mmd* and *Mmm*) and transect (Brandenburg and Bavaria). As described by Balard et al. 2020, we compared models including or excluding these predictors using likelihood-ratio tests and considered a predictor significant only in case of a significant increase in model likelihood. Our statistical framework compares nested models, starting from a linear model and adding parameters to increase complexity to reach nonlinear models. We only tested the significance of predictors (and this test, comparing one model with another nested within, accounts for the additional degrees of freedom in the more complex model). We perform inference on a few hypotheses and only one particular nonlinear hypothesis to avoid overfitting. All *p*-values were adjusted for false discovery rate (FDR) employing the Benjamini-Hochberg method.

To test for hybrid effects on richness, the Chao1 index, calculated as describe in section S1.5, was used as a measure of richness for the microbiome and predicted ARGs. The final set of ASVs and ARGs used for the analysis was selected based on potential ecologically relevant associations and significant effect on the community structure based on their Kleinberg's hub centrality scores. ASVs with Kleinberg's hub centrality above 0.1 score and with closeness centrality below 0.01 in the co-abundance network were tested. From the predicted ARGs, hub genes with Kleinberg's hub higher than 0.5 were chosen.

**Supplement 2. Permutational analysis of variance (PERMANOVA) for bacterial composition in natural populations of house mice (*Mus musculus*)**

|  | **Df** | **Sums Of Sqs** | ***F*-Model** | **R^2^** | **Pr(>*F*)** |
| --- | --- | --- | --- | --- | --- |
| *Locality* | 159 | 59.092 | 1.0031 | 0.32327 | 0.237 |
| *Year of collection* | 3 | 0.731 | 0.9867 | 0.00400 | 0.557 |
| ***Hybridicity*** | 1 | 0.510 | 1.3763 | 0.00279 | **0.047 *** |
| **Residuals** | 329 | 121.891 | - | 0.66682 | - |
| **Total** | 492 | 182.795 | - | 1.000 | - |

---

Df: Degrees of freedom, *F*-Model: *pseudo F*-test statistic, R^2^: Variance explained and *p* value based on 999 permutations.

**S**
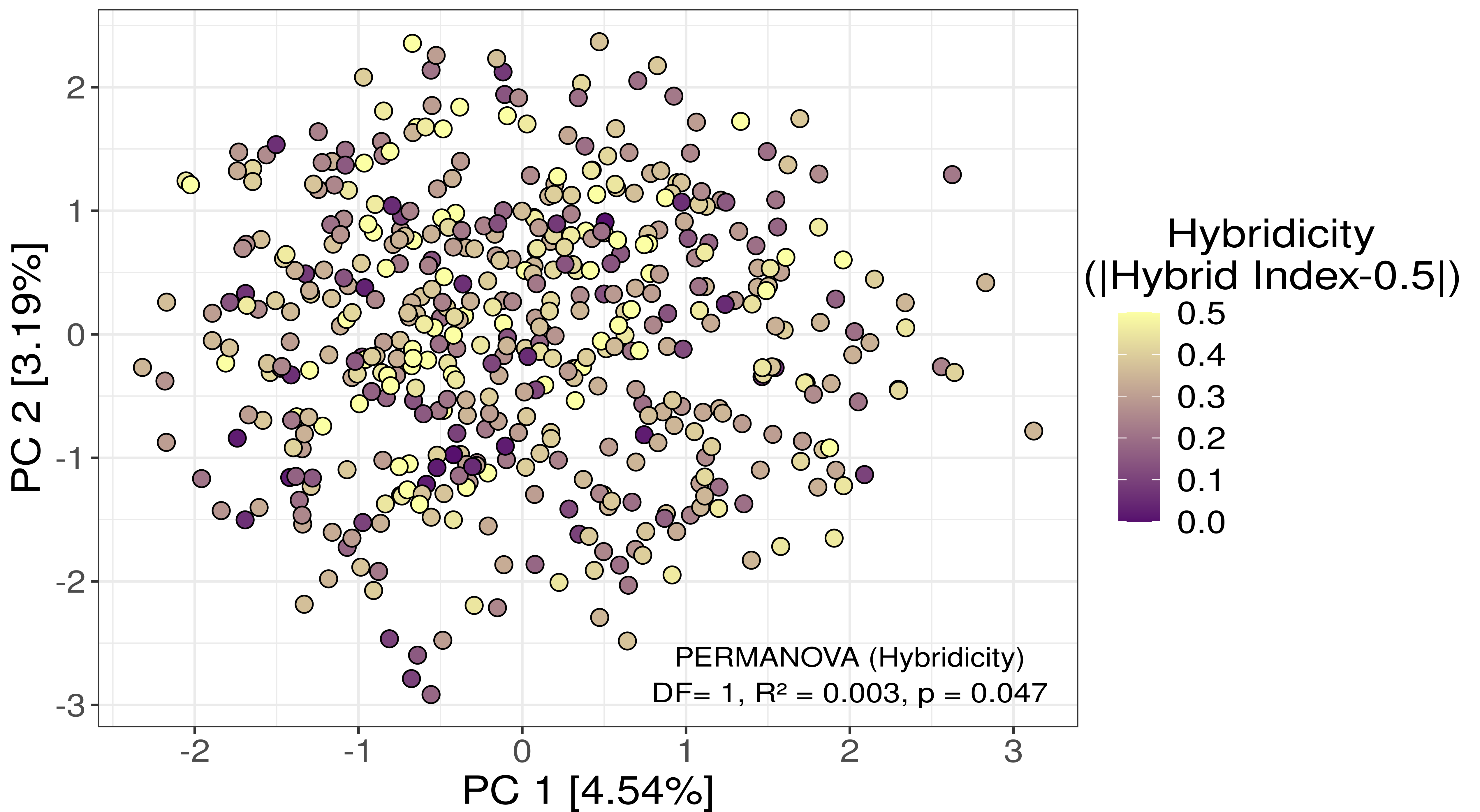
**upplementary Figure 2.1.** Principal component analysis (PCA) for ASV-level microbiome in house mice. Each dot represent an individual and colour code the hybridicity value for each mouse. Lack of clustering by host genetics or temporal and geographical features represented in two principal components PC1 and PC2 projection, which explained a low proportion of the total variance, though sampling location explained more than 30% of the microbial compositional variation (Bray-Curtis dissimilarity) but was not significant. Conversely, how hybridised a mouse is explained a smaller but significant proportion of these compositional changes.

| **Supplement 3. Chromosomal ARG content predicted from 16S rRNA gene amplicon data of house mouse colon content** | | | |
| --- | --- | --- | --- |
| **Kegg Ortholog** | **Gen** | **Description** | **Antibiotic type** |
| K03297 | emrE | small multidrug resistance pump | Multidrug |
| K03327 | mdtK | multidrug and toxic compound extrusions transporter | Multidrug |
| K03446 | emrB | translocase in the emrB -TolC efflux protein | Multidrug |
| K03543 | emrA | membrane fusion protein | Multidrug |
| K03585 | acrA | subunit of AcrA-AcrB-TolC multidrug efflux complex | Multidrug |
| K06159 | yojI | multidrug/microcin transport system ATP-binding/permease protein | Multidrug |
| K07552 | bcrC | undecaprenyl pyrophosphate phosphatase | Multidrug |
| K07786 | emrY | multidrug resistance protein | Multidrug |
| K07788 | mdtB | multidrug efflux pump | Multidrug |
| K07789 | mdtC | multidrug efflux pump | Multidrug |
| K07797 | emrK | multidrug resistance protein K | Multidrug |
| K07799 | mdtA | multidrug efflux system | Multidrug |
| K08152 | lmrP | multidrug resistance protein B | Multidrug |
| K08153 | blt | multidrug resistance protein | Multidrug |
| K08160 | mdfA | multidrug/chloramphenicol efflux transport protein | Multidrug |
| K08161 | mdtG | multidrug resistance protein | Multidrug |
| K08162 | mdtH | multidrug resistance protein | Multidrug |
| K08163 | mdtL | multidrug resistance protein | Multidrug |
| K08166 | mmr | methylenomycin A resistance protein | Multidrug |
| K08167 | smvA | multidrug resistance protein | Multidrug |
| K08168 | tetB | metal-tetracycline-proton antiporter | Tetracycline |
| K08169 | yebQ | multidrug resistance protein | Multidrug |
| K08170 | norB | multidrug resistance protein | Multidrug |
| K08217 | mef | macrolide efflux protein | Macrolide |
| K08218 | ampG | beta-lactamase induction signal transducer AmpG | Beta lactam |
| K09771 | ynfA | small multidrug resistance family-3 protein | Multidrug |
| K15547 | mdtO | multidrug resistance protein | Multidrug |
| K15549 | mdtN | multidrug efflux system | Multidrug |
| K15550 | mdtP | multidrug efflux system | Multidrug |
| K15974 | emrR | negative regulator of the multidrug operon emrRAB | Multidrug |
| K18094 | mexX | multidrug efflux system | Multidrug |
| K18095 | mexY | multidrug efflux pump | Multidrug |
| K18138 | acrB | multidrug efflux pump | Multidrug |
| K18139 | oprM | multidrug efflux system | Multidrug |
| K18142 | acrF | multidrug efflux pump | Multidrug |
| K18145 | adeA | multidrug efflux system | Multidrug |
| K18146 | adeB | multidrug efflux pump | Multidrug |
| K18147 | adeC | multidrug efflux system | Multidrug |
| K18149 | pbp5 | penicillin-binding protein | Beta lactam |
| K18214 | tetP_A | tetracycline resistant protein | Tetracycline |
| K18216 | steA | tetracycline resistant protein | Tetracycline |
| K18217 | steB | tetracycline resistant protein | Tetracycline |
| K18220 | tetM | ribosomal protection tetracycline resistance protein | Tetracycline |
| K18230 | tylC | macrolide transport system ATP-binding/permease protein | Macrolide |
| K18231 | msrA | macrolide transport system ATP-binding/permease protein | Macrolide |
| K18301 | mexL | transcriptional repressor | Tetracycline |
| K18302 | mexJ | multidrug efflux system | Multidrug |
| K18303 | mexK | multidrug efflux pump | Multidrug |
| K18321 | smeA | multidrug efflux system | Multidrug |
| K18322 | smeB | multidrug efflux pump | Multidrug |
| K18323 | smeC | multidrug efflux system | Multidrug |
| K18324 | acrD | multidrug efflux pump | Multidrug |
| K18325 | ramA | multidrug resistance transcriptional activator | Multidrug |
| K18326 | mdtD | multidrug resistance protein | Multidrug |
| K18887 | efrA | multidrug efflux pump | Multidrug |
| K18888 | efrB | multidrug efflux pump | Multidrug |
| K18889 | mdlA | multidrug efflux pump | Multidrug |
| K18890 | mdlB | multidrug efflux pump | Multidrug |
| K18891 | patA | multidrug efflux pump | Multidrug |
| K18892 | patB | multidrug efflux pump | Multidrug |
| K18893 | vcaM | multidrug efflux pump | Multidrug |
| K18898 | mdtE | multidrug efflux system | Multidrug |
| K18899 | mdtF | multidrug efflux pump | Multidrug |
| K18903 | oprC | multidrug efflux system | Multidrug |
| K18904 | nodT | multidrug efflux system | Multidrug |
| K18924 | ykkC | paired small multidrug resistance pump | Multidrug |
| K18925 | ykkD | paired small multidrug resistance pump | Multidrug |
| K18934 | lmrS | multidrug resistance protein | Multidrug |
| K18935 | sdrM | multidrug resistance protein | Multidrug |
| K18936 | mdeA | multidrug resistance protein | Multidrug |
| K18975 | qacEΔ1 | small multidrug resistance pump | Multidrug |
| K18989 | vexF | multidrug efflux pump | Multidrug |
| K18990 | vexE | multidrug efflux system | Quinolone |
| K19576 | norA | quinolone resistance protein | Multidrug |
| K19585 | oqxB | multidrug efflux pump | Multidrug |
| K19586 | oqxA | multidrug efflux system | Multidrug |
| K17836 | penP | beta-lactamase class A | Beta lactam |
| K17881 | aadB | Aminoglycoside 2-adenylyltransferase | Aminoglycoside |
| K00984 | aadA | aminoglycoside nucleotidyltransferase | Aminoglycoside |
| K19271 | catA | chloramphenicol O-acetyltransferase type A | Phenicol |
| K19272 | aph3-I | aminoglycoside 3'-phosphotransferase I | Aminoglycoside |
| K19273 | sat4 | streptothricin acetyltransferase | Aminoglycoside |
| K19274 | aph3-VI | Aminoglycoside 3-phosphotransferase VI | Aminoglycoside |
| K19299 | aph3-III | Aminoglycoside 3-phosphotransferase III | Aminoglycoside |
| K19300 | aph3-II | Aminoglycoside 3-phosphotransferase II | Aminoglycoside |
| K10673 | strA | Streptomycin 3-kinase | Aminoglycoside |
| K01467 | ampC | beta-lactamase class C | Beta lactam |
| K02171 | blaI | repressor of transcription of the blaZ/blaR1/blaI operon | Beta lactam |
| K02172 | blaR1 | upregulation of the blaZ/blaR1/blaI operon | Beta lactam |
| K17837 | blaB | Metallo-beta-lactamase class B | Beta lactam |
| K18698 | blaTEM | beta-lactamase class A TEM | Beta lactam |
| K18766 | blaZ | beta-lactamase class A BlaZ | Beta lactam |
| K18793 | blaOXA-23 | beta-lactamase class D OXA-23 | Beta lactam |
| K18794 | blaOXA-51 | beta-lactamase class D OXA-51 | Beta lactam |
| K19096 | blaCMY-2 | beta-lactamase class C CMY-2 | Beta lactam |
| K19097 | blaVEB | beta-lactamase class A VEB | Beta lactam |
| K19212 | blaOXA-63 | beta-lactamase class D OXA-63 | Beta lactam |
| K18824 | sul2 | dihydropteroate synthase type 2 | Sulfonamide |
| K04343 | strB | streptomycin 6-kinase | Aminoglycoside |
| K03862 | vanA | D-Ala-D-Ala ligase | Glycopeptide |
| K18552 | floR | florfenicol/chloramphenicol resistance protein | Phenicol |
| K18554 | cpt | chloramphenicol 3-O phosphotransferase | Phenicol |
| K18555 | mcbG | fluoroquinolone resistance protein | Quinolone |

**Supplement 4.**

**Supplement table 4.1. Transgressive phenotypes estimated by maximum likelihood for ARG and bacteria richness. 𝛼 represents the magnitude of the effect of hybridisation. Models including or excluding parental subspecies (*Mmd* and *Mmm*) and the two transect (Brandenburg and Bavaria) as predictors using likelihood-ratio tests and considered a predictor significant only in case of a significant increase in model likelihood**

| **Response** | **Predictors** | **𝛼**  **Hybrid effect** | **Richness *Mmd*** | **Richness *Mmm*** | ***padj -* value of hybrid effect** |
| --- | --- | --- | --- | --- | --- |
| ***Bacteria*** | | | | | |
| Richness  (Chao1 index) | Parental subspecies | 0.115 | 37.51 | 33.49 | 0.301 NS |
| Richness  (Chao1 index) | Parental subspecies and transect  **(Bavaria *N*=52)** | 1.573 | 29.35 | 15.05 | 0.002 ** |
|  | Parental subspecies and transect  **(Brandenburg *N*= 441)** | 0.097 | 37.73 | 34.42 | 0.398 NS |
| ***Antimicrobial resistance genes (ARG)*** | | | | | |
| Richness (Chao1 index) | Parental subspecies | 0.371 | 30.23 | 26.75 | 0.002 ** |
| Richness (Chao1 index) | Parental subspecies and transect  **(Bavaria *N*=52)** | 1.12 | 25.28 | 20.13 | 0.019 * |
|  | Parental subspecies and transect  **(Brandenburg *N*= 441)** | 0.331 | 30.51 | 27.16 | 0.007 ** |

***N*= Number of localities per transect**

**Supplement table 4.2. Transgressive phenotypes estimated by maximum likelihood for observed ARG and bacteria. 𝛼 represents the magnitude of the effect of hybridisation. Models including or excluding parental subspecies (*Mmd* and *Mmm*) and the two transect (Brandenburg and Bavaria) as predictors using likelihood-ratio tests and considered a predictor significant only in case of a significant increase in model likelihood**

| **Response** | **Predictors** | **𝛼**  **Hybrid effect** | **Richness *Mmd*** | **Richness *Mmm*** | ***padj -* value of hybrid effect** |
| --- | --- | --- | --- | --- | --- |
| ***Bacteria*** | | | | | |
| Richness  (Observed) | Parental subspecies | 0.100 | 35.30 | 32.00 | 0.371 NS |
| Richness  (Observed) | Parental subspecies and transect  **(Bavaria *N*=52)** | 1.491 | 28.183 | 15.10 | 0.004 ** |
|  | Parental subspecies and transect  **(Brandenburg *N*= 441)** | 0.076 | 35.50 | 32.87 | 0.567 NS |
| ***Antimicrobial resistance genes (ARG)*** | | | | | |
| Richness (Observed) | Parental subspecies | 0.363 | 30.08 | 26.62 | 0.002 ** |
| Richness (Observed) | Parental subspecies and transect  **(Bavaria *N*=52)** | 1.17 | 25.20 | 19.58 | 0.015 * |
|  | Parental subspecies and transect  **(Brandenburg *N*= 441)** | 0.317 | 30.36 | 27.07 | 0.007 ** |

***N*= Number of localities per transect**

**Supplement table 4.3. Transgressive phenotypes estimated by maximum likelihood for ARG and bacteria diversity (Shannon index). 𝛼 represents the magnitude of the effect of hybridisation. Models including or excluding parental subspecies (*Mmd* and *Mmm*) and the two transect (Brandenburg and Bavaria) as predictors using likelihood-ratio tests and considered a predictor significant only in case of a significant increase in model likelihood**

| **Response** | **Predictors** | **𝛼**  **Hybrid effect** | **Richness *Mmd*** | **Richness *Mmm*** | ***padj -* value of hybrid effect** |
| --- | --- | --- | --- | --- | --- |
| ***Bacteria*** | | | | | |
| Diversity  (Shannon index) | Parental subspecies | 0.027 | 2.92 | 2.79 | 0.732 NS |
| Diversity  (Shannon index) | Parental subspecies and transect  **(Bavaria *N*=52)** | 0.576 | 2.63 | 2.07 | 0.024 * |
|  | Parental subspecies and transect  **(Brandenburg *N*= 441)** | 0.005 | 2.937 | 2.830 | 0.925 NS |
| ***Antimicrobial resistance genes (ARG)*** | | | | | |
| Diversity  (Shannon index) | Parental subspecies | 0.102 | 2.63 | 2.54 | 0.041 * |
| Diversity  (Shannon index) | Parental subspecies and transect  **(Bavaria *N*=52)** | 0.405 | 2.41 | 2.30 | 0.042 * |
|  | Parental subspecies and transect  **(Brandenburg *N*= 441)** | 0.073 | 2.65 | 2.56 | 0.059 NS |

***N*= Number of localities per transect**

**S**
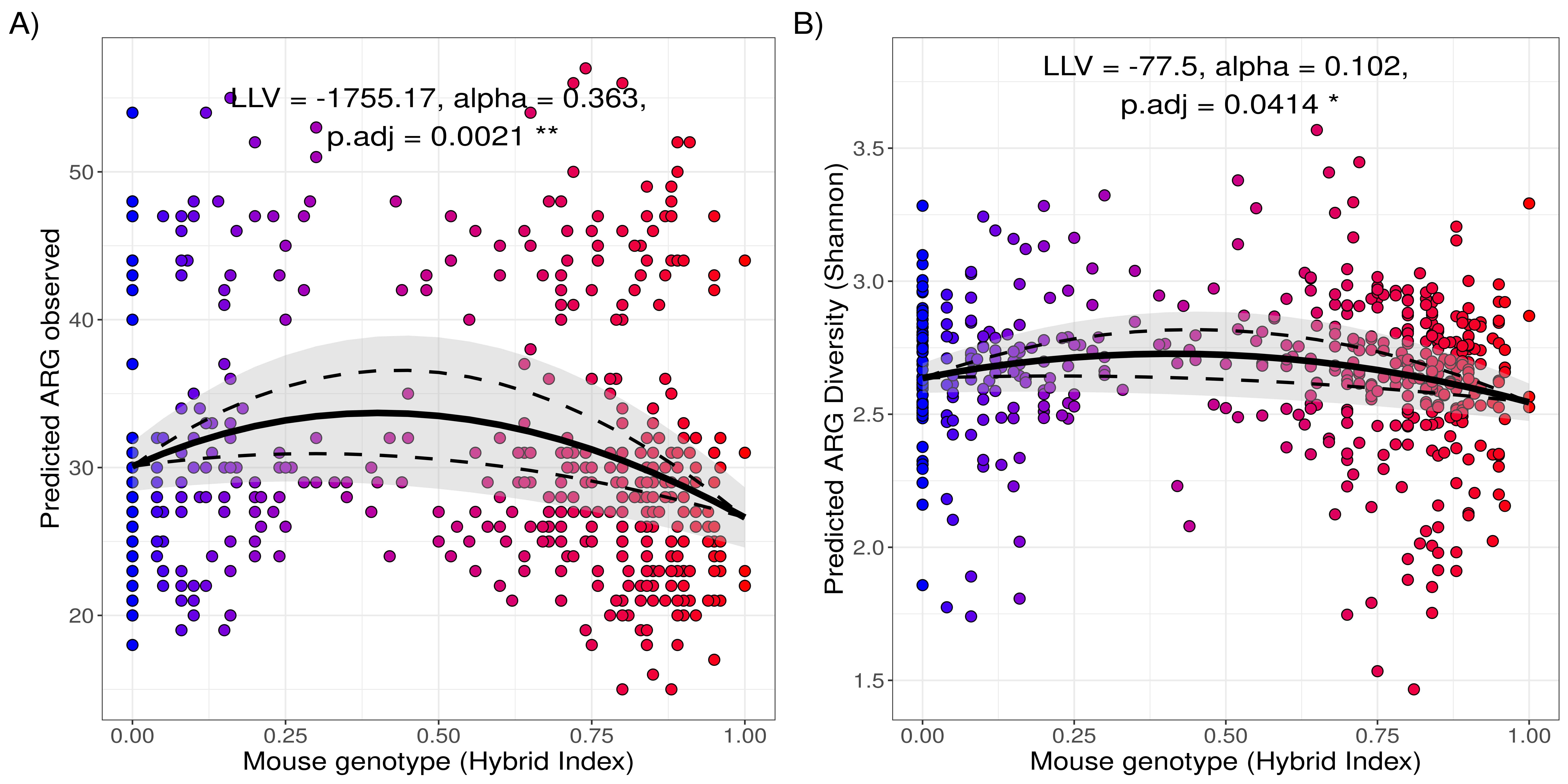
**upplement Figure S4.4. Hybrid effect on observed ARG richness and diversity confirm the effect observed for Chao1 ARG richness.** ARG observed richness predictions and ARG diversity (Shannon index) were compared across a gradient of *Mus musculus* genotypes (HI), ranging from 0 (pure *M. m. domesticus*, in blue) to 1 (pure *M. m. musculus*, in red), to (i) test hybrid effect on alpha diversity and (ii) detect differences on alpha diversity between parental subspecies, transects or both. The observed richness **(A)** and diversity **(B)** of predicted ARGs increased towards the centre of the hybrid zone, supporting a hybrid (transgressive) effect on ARGs (Observed ARG, LLV= -1755.17, 𝛼= 0.363, *padj=* 0.0021; Shannon index, LL= -77.5, 𝛼= 0.102, *padj=* 0.0414).


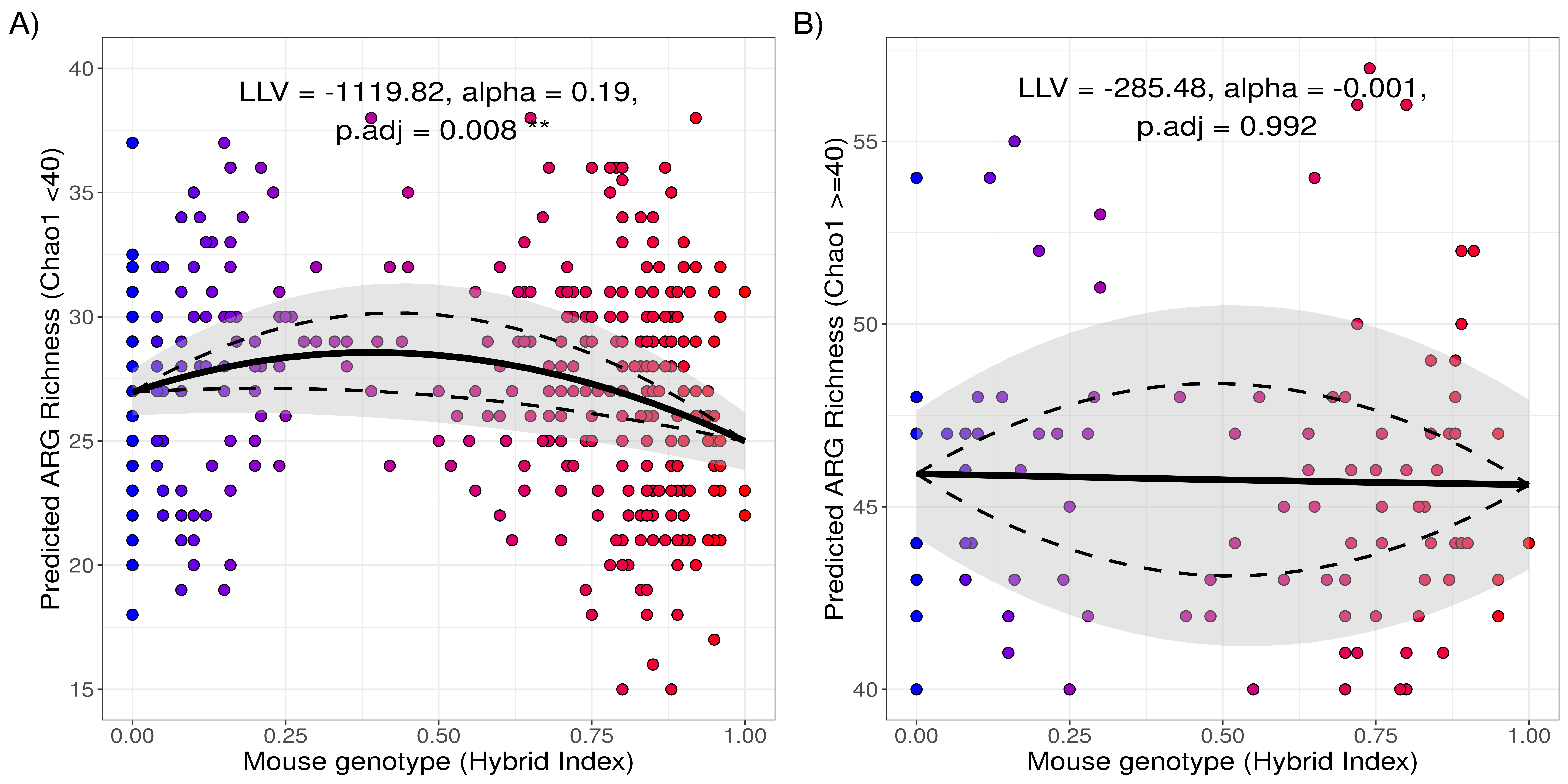


**Supplement Figure S4.5.** **Hybrid effect is robust for those individuals within the main level of ARG richness (Chao1 < 40).** The bimodal distribution of ARG richness does not impact the observed hybrid effect on ARG richness differs between those with “high” richness (Chao1 ≧40) versus those with "low/regular" ARG richness (Chao1 <40). **A)** The hybrid effect in the majority of the samples with "low/regular " ARG richness individuals remained a significant signal (N= 390, LLV= -1119.82, α= 0.191, *p.adj*= 0.008). **B)** The effect in the minority of individuals with "high" ARG richness was not detected (N= 103, LLV= -289.48, α= -0.001, *p.adj*= 0.992).

**Supplement 5. Analysis of variance in predicted ARG alpha diversity explained by host, microbiome and sampling derived factors. Significance for each predictor was determined by nested linear model comparison by** **likelihood ratio test (LRT) and the variance explained (Var. exp) by** **the coefficient of determination (*R*^2^*100)**

| **Response/**  **Predictor** |  | ***ARG Richness***  ***(Chao1 Index)*** | | | ***ARG Diversity***  ***(Shannon Index)*** | | | ***ARG Evenness***  ***(Simpson Index)*** | | |
| --- | --- | --- | --- | --- | --- | --- | --- | --- | --- | --- |
|  | **DF** | **Var. exp (%)** | **Chisq** | ***p*-**  **value** | **Var. exp (%)** | **Chisq** | ***p*-**  **value** | **Var. exp (%)** | **Chisq** | ***p*-**  **value** |
| ***Hybridicity*** | 1 | 3.41 | 7.89 | **0.005**  ****** | 2.82 | 2.77 | 0.096 | 7E-4 | 0.015 | 0.903 |
| ***Year of collection*** | 3 | 2.54 | 0.14 | 0.931 | 1.36 | 1.16 | 0.56 | 2.5 | 1.96 | 0.376 |
| ***Locality*** | 159 | 63.6 | 208.42 | **0.005**  ****** | 61.45 | 210.04 | **0.004**  ****** | 59.48 | 219.25 | **0.001**  ****** |
| ***Bacteria richness*** | 1 | 6.47 | 37.69 | **8.3E-10**  ******* | 0.96 | 27.85 | **1.3E-7**  ******* | 2.44 | 0.24 | 0.625 |
| ***Firmicutes abundance*** | 1 | 0.67 | 13.87 | **1.97E-4**  ******* | 4.66 | 48.45 | **3.4E-12**  ******* | 2.96 | 18.42 | **1.8E-05**  ******* |
| ***Bacteroidetes abundance*** | 1 | 2.61 | 5.17 | **0.023**  ***** | 1.10 | 0.41 | 0.524 | 0.09 | 1.3 | 0.254 |
| ***Proteobacteria***  ***abundance*** | 1 | 10.75 | 50.33 | **1.3E-12**  ******* | 18.74 | 84.7 | **2.2E-16**  ******* | 1.51 | 7.44 | **0.007**  ****** |
| ***Residuals*** |  | 9.95 |  |  | 8.92 |  |  | 31.01 |  |  |

**Supplement 6. Permutational analysis of variance (PERMANOVA) for predicted ARG composition in the microbiome from natural populations of house mice (*Mus musculus*)**

|  | **Df** | **Sums Of Sqs** | ***F*-Model** | **R^2^** | **Pr(>*F*)** |
| --- | --- | --- | --- | --- | --- |
| *Locality* | 158 | 23.140 | 1.020 | 0.325 | 0.057 |
| *Year of collection* | 3 | 0.214 | 0.749 | 0.003 | 0.576 |
| *Hybridicity* | 1 | 0.580 | 4.064 | 0.008 | **0.012*** |
| **Residuals** | 329 | 46.931 | - | 0.660 | - |
| **Total** | 492 | 71.107 | - | 1.0000 | - |

---

Df: Degrees of freedom, *F*-Model: *pseudo F*-test statistic, R^2^: Variance explained and *p* value based on 999 permutations.

**Supplement 7. General comparison of ARG profiles based on PICRUSt2 and shotgun metagenomics.**

**
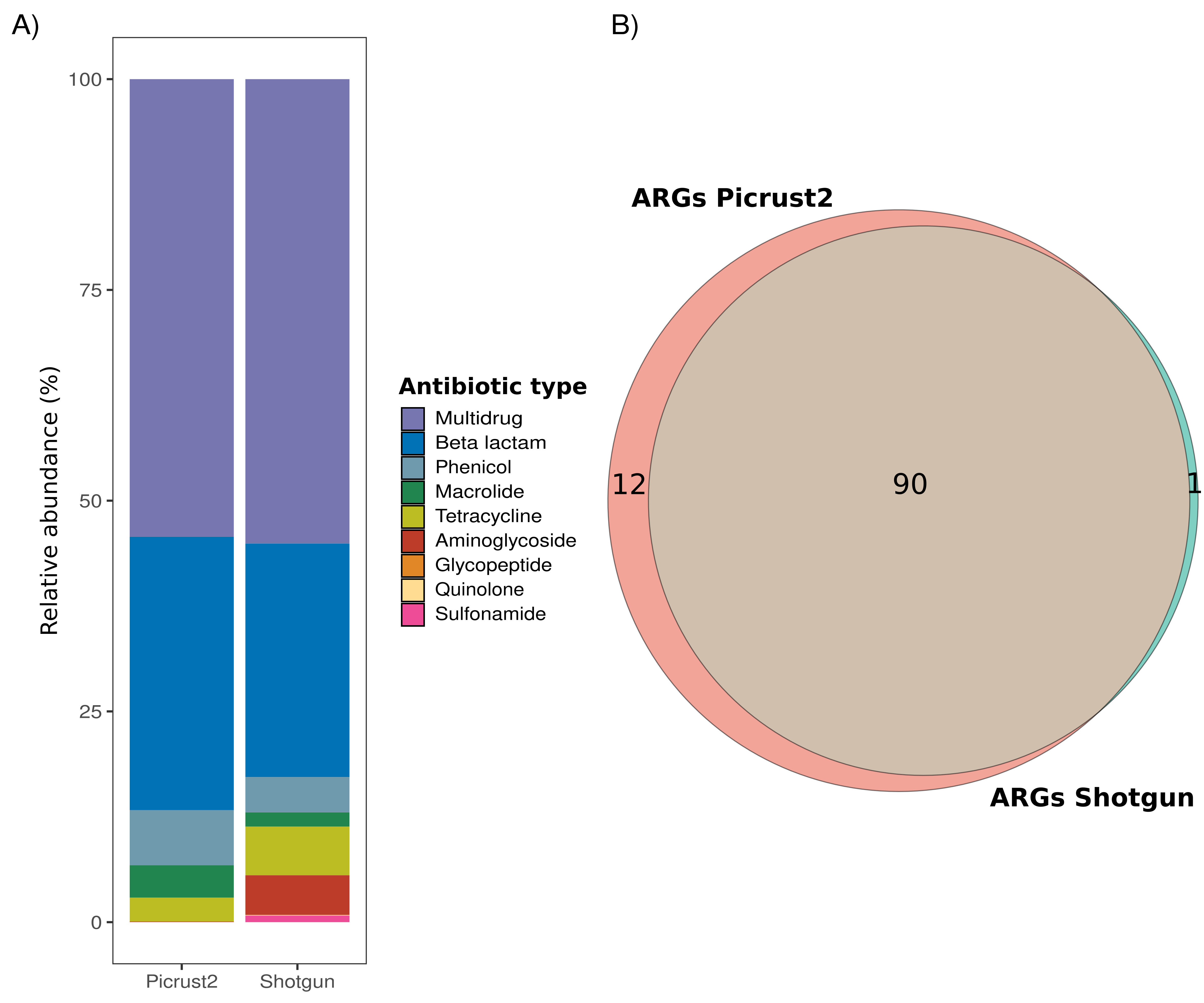
**

**Supplement Figure S7.1. Congruent ARG profiles in colon content DNA based on PICRUSt2 prediction and shotgun assessment.** Shotgun ARG profiles were assessed for an independent data set of colon-content DNA samples from 6 mice. ARG annotation was done using the Global Microbial Gen Catalog v1 and obtaining Kegg Orthologues corresponding to ARGs to compare directly against the profiles estimated by PICRUSt2. A) Mean relative abundance of ARGs by the antimicrobial target type. PICRUSt2 prediction underestimated the abundance of ARGs targeting aminoglycosides and sulfonamides. However, it recapitulated the abundance of other drugs, such as multidrug and beta-lactam ARGs. B) PICRUSt2 and shotgun ARG profiles share the majority of the genes. Even when PICRUSt2 underestimates the mean abundance for particular ARG types, it overall predicts more genes than the shotgun assessment.

**
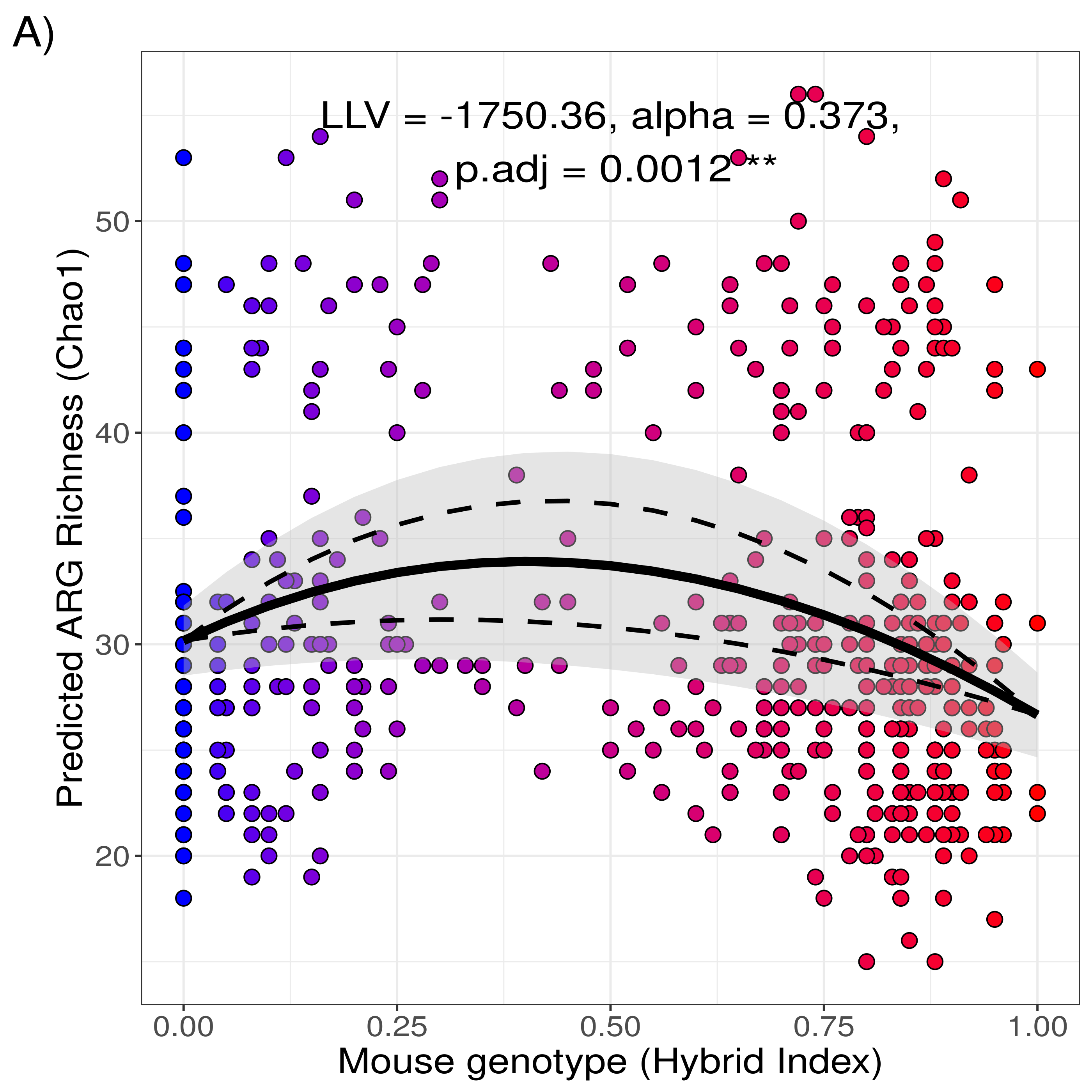
**

**Supplement Figure S7.2.** **Hybrid effect on ARG richness predicted with Picrust2 is robust when only ARGs detected with metagenomics are considered.** The ARG richness in hybrids remains higher when considering only ARGs detected by metagenomics. The richness of predicted only considering ARGs detected by Picrust2 and shotgun metagenomics (90 ARGs) increased towards the centre of the hybrid zone (LLV= -1750.63, α= 0.373, *p.adj*= 0.0012).
